# Anthropogenic *Blastocystis* from Drinking Well and Coastal Water in Guinea-Bissau (West Africa)

**DOI:** 10.3390/microorganisms13030620

**Published:** 2025-03-07

**Authors:** Sara Gomes-Gonçalves, Ana Machado, Adriano Bordalo, João R. Mesquita

**Affiliations:** 1 ICBAS—School of Medicine and Biomedical Sciences, Porto University, 4050-313 Porto, Portugal; 2 CIIMAR-Interdisciplinary Centre of Marine and Environmental Research, University of Porto, Novo Edifício do Terminal de Cruzeiros do Porto de Leixões, Avenida General Norton de Matos, S/N, 4450-208 Matosinhos, Portugal; 3 Centro de Estudos de Ciência Animal (CECA), Instituto de Ciências, Tecnologias e Agroambiente (ICETA), Universidade do Porto (UP), Rua D. Manuel II, Apartado 55142, 4051-401 Porto, Portugal; 4 Associate Laboratory for Animal and Veterinary Science (AL4AnimalS), 1300-477 Lisboa, Portugal

**Keywords:** *Blastocystis*, waterborne parasites, Guinea-Bissau water quality, qPCR, drinking water contamination

## Abstract

This study provides the first comprehensive investigation of *Blastocystis* sp. contamination in the drinking well and coastal water sources in Guinea-Bissau, a region grappling with severe water quality challenges. Of the forty-five water samples analyzed (34 well and 9 coastal water sources), *Blastocystis* sp. was detected in five (11%, 95% CI: 3.71–24.05) of the wells, which serve as a critical and primary drinking source for local communities. The detection of human-associated *Blastocystis* sp. subtype (ST)2 and ST3 raises concerns about the potential of fecal contamination as a transmission route for *Blastocystis* sp., underscoring the public health risks associated with an inadequate WASH (water, sanitation, and hygiene) infrastructure. These findings highlight the urgent need for improved water management and further research on waterborne parasitic infections in resource-limited settings.

## 1. Introduction

Access to clean drinking water is recognized as a fundamental human right and is essential for both individual survival and overall well-being. Ensuring that all individuals, regardless of socio-economic status, have reliable access to potable water is crucial for public health and sustainable development. The availability of safe drinking water directly impacts hygiene, sanitation, and overall quality of life, particularly in regions where waterborne diseases are prevalent. Inadequate access to clean water contributes to increased morbidity and mortality rates, especially among vulnerable populations such as children, the elderly, and immunocompromised individuals. This pressing issue underscores the need for targeted interventions, policy reforms, and investments in water infrastructure to mitigate the risks associated with contaminated water sources. Sustainable Development Goal (SDG) 6.1 emphasizes the need to provide universal and equitable access to safe and affordable drinking water [1,2]. However, more than 2.2 billion people, roughly one-fourth of the world population, lack reliable access to safe water, particularly in low-income countries [3]. This scarcity not only poses several health risks but also adversely impacts the quality of life for millions of people [4]. 

Guinea-Bissau exemplifies this challenge, ranking among the 20 countries with the poorest water quality. According to the World Population Review, only 24% of its population has access to safely managed drinking water services [5]. Furthermore, the United Nations Development Program lists Guinea-Bissau as the 11th poorest country globally [6]. The country faces significant challenges due to inadequate investment in water sanitation infrastructure, inefficient resource management, and limited governmental oversight. Many rural and urban communities rely on surface water or shallow wells, both of which are highly susceptible to contamination from human and animal waste [7,8] The severe lack of water, sanitation, and hygiene (WASH) infrastructure in this sub-Saharan country is alarming, with over 80% of the shallow, hand-dug wells, used by the majority of the population to suppress daily needs, contaminated with fecal material and exhibiting acidic pH levels [7]. Acidic pH can affect the taste of water and reduce its acceptability, and long-term consumption can contribute to tooth erosion and digestive problems [8]. These conditions create an environment conducive to the spread of intestinal protozoa including *Blastocystis*, which is primarily transmitted through contaminated food or water. 

Research on intestinal protozoa in Guinea-Bissau has mostly focused on well-known pathogens such as *Cryptosporidium*, *Giardia duodenalis*, and *Entamoeba histolytica* [9,10]. In contrast, the research on *Blastocystis* remains limited, with only two studies conducted in the country, and only one regarding its presence in humans [11,12]. Compounding this issue, the Guinea-Bissau healthcare system is fragile and not universally accessible, with a high burden of diseases such as malaria, diarrhea, respiratory infections, HIV, and malnutrition. In 2019, diarrheal diseases were the third leading cause of death in the country, with 702,974 reported cases [8]. Studies have shown that intestinal parasitic infections can worsen immunosuppression in individuals with HIV/AIDS, increasing their vulnerability to opportunistic infections [13]. As a result, diarrheal diseases, which the World Health Organization ranks among the top 10 causes of death in low-income countries, remain a pressing concern [14].

*Blastocystis* is a stramenopile of uncertain pathogenicity, despite being known about for over a century due to the frequent occurrence of asymptomatic infections [15,16]. Latent infections often go unnoticed in healthy individuals but can pose risks to immunocompromised patients, especially in low-income countries with limited clean water and healthcare. [17]. With an estimated global infection rate between one and two billion people, *Blastocystis* is the most common gastrointestinal parasite worldwide [18,19]. The transmission of *Blastocystis* occurs primarily through the fecal–oral route, either by direct contact with infected individuals or the indirect ingestion of contaminated food or water [20,21]. At the moment, *Blastocystis* comprises at least 44 identified subtypes (ST1–ST17, ST21, and ST23–ST48), of which only 16 are considered zoonotic (ST1-5, ST10, ST12, ST14, ST16, ST23, ST35, and ST41) [22,23]. Among these, ST1–ST4 account for approximately 90% of anthropogenic infections, as they are primarily associated with human-to-human transmission [24]. These subtypes have been linked to a range of gastrointestinal symptoms, from mild discomfort to severe diarrhea [17]. Despite its global prevalence, *Blastocystis* remains understudied in many regions, including West Africa, where limited epidemiological data hinder effective public health intervention. While less common subtypes appear to have a lower prevalence in human populations, their potential public health impact should not be underestimated. The last systematic review and metanalysis on *Blastocystis* in water sources showed that the prevalence in drinking and surface water was 19.1% and 17.6%, respectively, including zoonotic subtypes ST1-ST4, ST6, ST8, and ST10. These findings highlight the need to improve the cleanliness and quality of water sources and promote public health awareness [25].

The lack of reliable access to safe drinking water in Guinea-Bissau contributes to the high prevalence of diarrheal diseases and facilitates the spread of waterborne parasites like *Blastocystis*, which rely on contaminated water as a primary transmission route. Despite the known risks of waterborne parasites in resource-limited settings, studies on *Blastocystis* contamination in drinking water sources are scarce, particularly in West African countries like Guinea-Bissau, where research efforts have primarily focused on bacterial and viral pathogens. This study is the first to investigate the presence of *Blastocystis* in both the drinking well water and coastal water sources in Guinea-Bissau, addressing a gap in the literature. By assessing the presence of *Blastocystis* and its zoonotic subtypes in various water sources, this study provides epidemiological data that can inform public health policies and water management strategies. These findings offer new insights into water contamination in the region, particularly highlighting the presence of zoonotic *Blastocystis* subtypes, which may pose public health risks.

## 2. Material and Methods

### 2.1. Sampling

A convenience sampling approach was used from the previous studies performed by Machado, A., and Bordalo, A. A (2014) [26] and Machado, A., and Bordalo, A. A. (2016) [27]. The sample selection focused on high-risk water sources, particularly shallow drinking water wells fitted with a bucket and rope to withdraw water, with and without any wall isolation or well cover protection, making them highly vulnerable to fecal contamination, especially during the wet season.

To maximize the assessment of anthropogenic influence, sampling was primarily conducted during the wet season, when contamination risks are potentially the highest [7]. The sampling sites were located in the vicinity of pit latrines, a common practice among the local population that increases the likelihood of fecal contamination from human influence. Nine primary wells, which supply most of Bolama Island (11.579 N; 15.472 W) with domestic water, were surveyed in 2010 during both the dry (*n* = 10) and wet seasons (*n* = 24). Additionally, surface water samples were collected from three coastal sites known to be impacted by urban runoff and human activity (*n* = 11).

**Figure 1:**
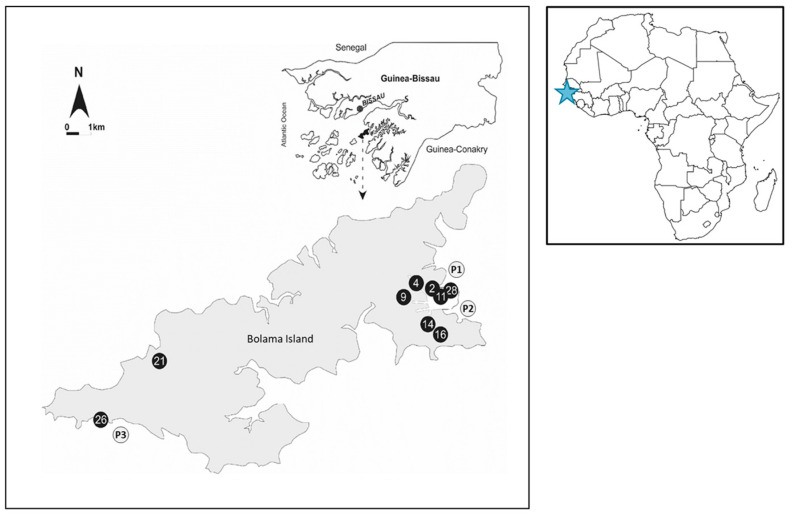
Geographical distribution of the sampling site. Sample sites described by black circles correspond to the well samples while the gray circles correspond to the coastal samples. The blue star marks the geographical location of Guinea-Bissau within the African continent.

The exact position of each surveyed site was recorded by means of GPS (Magellan600, Magellan, Santa Clara, CA, USA) (Figure 1). 

All samples were kept in the dark in refrigerated ice chests and processed within 4 h of collection at a field laboratory similar to the one described in Bordalo and Savva-Bordalo (2007) [28]. Detailed information about the sampling area and sampling site characterizations can be found in Machado and Bordalo (2014) [26]. 

### 2.2. DNA Extraction

For DNA extraction, water samples ranging from 100 to 400 mL were filtered through sterile cellulose nitrate membranes (0.2 μm pore size, 47 mm diameter, Whatman, Maidstone, UK) and stored at −20 °C until nucleic acid extraction. Environmental DNA was isolated using a modified CTAB (bromide-polyvinylpyrrolidone-β-mercaptoethanol) following the method of Dempster et al. (1999), as detailed by Barrett et al. (2006) [29,30]. DNA quality was assessed on an agarose gel and quantified using a Qubit fluorometer with the Quant-iT dsDNA assay (Invitrogen, Carlsbad, CA, USA).

### 2.3. Molecular Detection of Blastocystis sp. and Sanger Sequencing 

The detection of *Blastocystis* sp. was performed by a Real-time Polimerase Chain Reaction (qPCR) targeting the small ribosomal subunit (SSU) rRNA gene to amplify it to approximately 300 bp as described in [31]. The qPCR reactions were performed in a total volume of 20 µL using the Xpert Fast SYBR Uni (GRiSP^®^, Porto, Portugal), following the manufacturer’s guidelines. The primer pair BL18SPPF1 and BL18SR2PP was used for amplification. The thermal cycling conditions consisted of an initial denaturation at 95 °C for 5 min, followed by 45 cycles at 95 °C for 5 s and annealing at 68 °C for 20 s, during which time fluorescence data were recorded. After the completion of the PCR cycles, a melting curve analysis was conducted (1 min at 68 °C, followed by 15 s at 95 °C) to differentiate the specific amplicons from the non-specific products, with the melting temperature (Tm) determined at the peak of the curve. The qPCR assays were carried out using a CFX Connect Real-Time PCR Detection System (Bio-Rad, Hercules, CA, USA), and the results were analyzed with CFX Maestro 1.0 Software version 4.0.2325.0418 (Bio-Rad, Hercules, CA, USA). Positive controls (positive samples obtained in a former study [32]), and a no-template control (a PCR mix and RNAse-free water instead of a DNA template) were used. 

The positive samples analyzed by the qPCR that matched the expected melting temperatures were purified and directly sent for Sanger sequencing. The chromatograms that showed a single *Blastocystis* infection were analyzed by the Basic Local Alignment Search Tool (BLAST; https://blast.ncbi.nlm.nih.gov/ accessed on 15 January 2025) for *Blastocystis* confirmation.

### 2.4. Oxford Nanopore Technology (ONT) Sequencing

The samples displaying double peaks on the chromatograms, indicative of mixed infections, were selected for Next Generation Sequencing using Oxford Nanopore Technology. The high-throughput PromethION 24 platform was employed and optimized for generating extensive sequencing data. To enhance the read accuracy, the R10.4.1 flow cell was used, benefiting from its advanced pore design and refined chemistry. Library preparation was performed with the Native Barcoding Kit 96 V14 (SQK-NBD114.96), allowing for multiplexed sequencing while preserving the native DNA structure, which is ideal for specialized studies such as epigenetics and long-read analyses.

The raw FASTQ reads were basecalled using ont-dorado-for-promethion v7.4.12 in super-accurate mode, ensuring a minimum Q-score of 10. Adapter and barcode trimming were executed through MinKNOW (https://github.com/nanoporetech/minknow_api accessed on 15 January 2025). The subsequent analyses were conducted via a custom pipeline, which included processing raw sequences, trimming them to lengths of 200–350 bp, filtering, clustering, checking for chimeras, polishing, and subtyping using BLAST against a curated database.

### 2.5. Phylogenetic Analysis 

A phylogenetic analysis based on the full-length SSU rRNA gene sequences was performed to ensure the correct assignment of subtypes. Reference sequences were sourced from the curated https://entamoeba.lshtm.ac.uk/ref.blasto.txt (accessed on 15 January 2025) database, ensuring reliable comparisons. Sequence alignment was conducted using MAFFT v7.490, applying the L-INS-i algorithm, which is optimized for datasets with large numbers of sequences and complex structural variations. Phylogenetic tree construction was carried out with the IQ-TREE (version 2.4.0). The analysis included 1000 bootstrap replicates to assess the statistical support of the branching patterns, using the Kimura 2-parameter model with invariant sites and a gamma distribution (K2P + I + G4). *Proteromonas lacertae*, a commensal flagellate commonly found in reptiles and amphibians, was selected as the outgroup because of its previously established close phylogenetic connection to *Blastocystis* found in earlier research [22,33,34]. Further annotations, including taxonomic labels and subtype-specific features, as well as graphical enhancements for publication-quality figures, were added using the Interactive Tree of Life (iTOL) platform (https://itol.embl.de/ accessed on 20 January 2025) [35].

### 2.6. Statitical Analysis

The presence of *Blastocystis* sp. was evaluated by determining the ratio of positive samples to the total number of samples analyzed, along with the associated 95% confidence interval (95% CI). Data processing and the initial analysis were conducted using Microsoft Excel^®^ for Microsoft 365 MSO (Redmond, WA, USA) (version 2312 Build 16.0.17126.20132, 64-bit).

## 3. Results

In this study, a total of 45 water samples were collected from well water and coastal water sources in Guinea-Bissau to investigate the presence of *Blastocystis* sp. Among these, five samples (11%; 5/45; 95% CI: 3.71–24.05) tested positive for *Blastocystis* sp., all of which originated from three different wells. In contrast, none of the coastal water samples contained detectable *Blastocystis* sp., suggesting a possible difference in contamination sources between the well and coastal waters. This discrepancy highlights the urgent need for targeted interventions aimed at improving the water quality in well sources, particularly in regions where access to safe drinking water is limited. Additionally, it emphasizes the need for the regular monitoring of both well and coastal waters to identify potential contamination sources. When considering only the well water samples (n = 34), the occurrence rate was 15% (5/34; 95% CI: 4.95–31.06), highlighting that well water could serve as a potential transmission route for this parasite. These findings are concerning, as they indicate a significant risk for the local populations who depend on these wells for their daily water needs.

**Figure 2:**
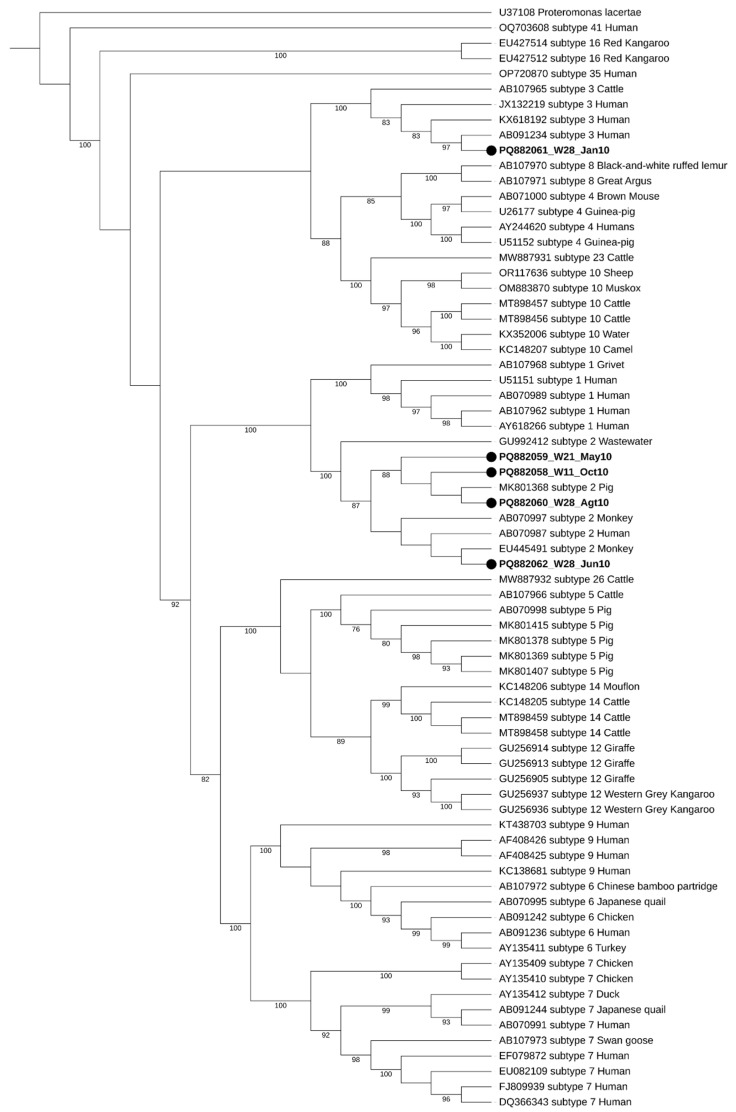
Maximum-likelihood phylogenetic tree of *Blastocystis* sp. STs based on the comparison of SSU rRNA gene sequences. The tree was generated using IQ-TREE with the K2P + I + G4 substitution model and 1000 bootstrap replicates. *Proteromonas lacertae* was used as a suited outgroup. The sequences obtained in this study are highlighted in bold, labeled with their accession number, well number (W11, W21, and W28), and the date of collection, respectively. Bootstraps below 75% are not displayed.

During the initial screening, two well water samples showed double peaks in their chromatograms, raising the possibility of mixed Blastocystis sp. subtypes (STs). However, the subsequent analysis using Oxford Nanopore Technology (ONT) sequencing confirmed that these samples were negative for *Blastocystis* sp. This highlights the complexity of accurately identifying parasites in environmental samples, as mixed infections can lead to ambiguous results. Regarding seasonal distribution, two of the five positive samples were collected during the dry season (W28_Jan10 and W21_May10), while the remaining three were obtained in the wet season (W28_Jun10, W28_Agt10, and W11_Oct10), as illustrated in Figure 2. The seasonal variations in contamination levels underscore the impact of rainfall and flooding on water quality, suggesting that increased rainfall may exacerbate contamination risks. The subtypes identified in this study were ST2 and ST3, with ST2 being the most frequently detected, appearing in four out of the five positive samples.

Further water quality analyses, detailed in previous studies [26,27], confirmed that all the sampled water sources, regardless of season, exhibited significant fecal contamination. The levels of contamination exceeded the legal limits set by the European Union (EU) and the World Health Organization (WHO), reaching concentrations as high as 5 Log. These alarming levels of contamination highlight the critical need for enhanced sanitation practices and public health education to reduce exposure to waterborne pathogens. Addressing these issues is vital for protecting the health of the local population and ensuring access to safe drinking water. This high degree of fecal pollution indicates there are potential health risks associated with the consumption or use of these water sources.

## 4. Discussion

This study investigated the presence of *Blastocystis* in the drinking well water and coastal water sources in Guinea-Bissau and provides compelling evidence for the presence of *Blastocystis* sp. in the well water sources in Guinea-Bissau, with anthropogenic subtypes (ST2 and ST3) detected in multiple samples. To our knowledge, this represents the first investigation of *Blastocystis* sp. in these water sources, marking a major step toward understanding the transmission of this protozoan in resource-limited settings. The finding of contamination in three out of the nine sampled wells raises significant public health concerns, particularly given that local communities depend on these wells for their drinking water. These shallow hand-dug wells, which are less than 15 m deep, present a greater contamination risk compared to the deeper boreholes (more than 20 m) that are mechanically drilled and equipped with solar or electric pumps. During the wet season, these wells are replenished, and their shallow depth facilitates increased infiltration and the percolation of surface water. Moreover, open defecation practices and roaming domestic animals could serve as additional sources of contamination, as the proximity of existing latrines to the wells is often insufficient to prevent the entry of human pathogenic microorganisms. [7,26]. Furthermore, a previous study conducted in a neighboring country found that the individuals consuming water from drilled wells had twice the risk of *Blastocystis* infection compared to those not consuming drilled well water [36]. 

The identification of *Blastocystis* sp. in drinking water sources underscores the potential for zoonotic and human-to-human transmission through fecal-contaminated water. The presence of the subtypes associated with humans further implicates poor sanitation as a key driver of contamination [37,38,39]. These findings are consistent with previous reports as the same STs found in this study were found in humans within different African countries with inadequate water infrastructure [40,41,42,43,44,45]. Although fully understanding the pathogenicity of *Blastocystis* sp. remains a challenge, it has been associated in some cases with symptoms ranging from mild discomfort to severe diarrhea, rectal bleeding, anorexia, nausea, vomiting, fever, and irritable bowel syndrome [17,46,47]. These infections can be particularly concerning for immunocompromised individuals, such as those living with HIV or suffering from malnutrition. Guinea-Bissau has the second-highest HIV prevalence in West Africa, with 5.3% of the reproductive-age population affected, and malnutrition rates exceed 28%, according to UNICEF and the World Food Program [48,49]. This demographic vulnerability may amplify the potential health impact of *Blastocystis* sp., as the parasite could exacerbate the existing health issues under conditions of compromised immunity. Recent studies have shown that intestinal parasitic infections can worsen the overall health outcomes for individuals with HIV/AIDS, leading to increased morbidity. [50,51]. Despite the lack of literature on the presence of *Blastocystis* in Guinea-Bissau, a previous study reported the presence of *Blastocystis* in HIV-positive individuals with chronic diarrhea [11]. 

For the wells, only three out of the eight sampled sites tested positive for *Blastocystis* sp. However, the detection of *Blastocystis* sp. in three of the samples from the well serving water to the primary school of Sanzala (W28) is particularly troubling. The proximity of the latrines frequented by the students may explain the continuous presence of anthropogenic *Blastocystis* in both the dry and wet seasons. More than half of the recorded deaths due to diarrheal diseases were from children under five. Due to their underdeveloped immune systems, children are particularly vulnerable to parasitic infections, and exposure to contaminated water can lead to severe health consequences [52,53,54]. This is particularly worrisome as the well is used by schoolchildren, a group more vulnerable to parasitic infections including protozoa [55,56]. The other two positive sites were in the wells in Sanzala and Ponta Doce Balanta (W11 and W21, respectively). The presence of *Blastocystis* in well water is scarcely reported in the literature. To the best of our knowledge, only two studies have investigated its occurrence in well water, one in Malaysia [57] and another in Mexico [58], with each reporting ST1 and ST3 prevalence rates of 100% (5/5) and 0% (0/3), respectively, aligning with the results in this study regarding the presence of anthropogenic STs.

These findings underscore the urgent need for targeted interventions to protect vulnerable populations, particularly school-aged children, who are at a higher risk of infection. Recommendations for public health initiatives include periodic deworming campaigns with appropriate drugs against *Blastocystis* sp., such as Nitazoxanide, which could be integrated into the national health programs that currently focus on Mebendazole or Albendazole. The latest campaign occurred in December 2024, illustrating the ongoing efforts to combat parasitic infections. 

Interestingly, while fecal contamination was widespread, only 15% of the samples tested positive for *Blastocystis* sp. This variability may reflect differences in water usage, sanitation practices, or environmental factors such as rainfall and soil permeability. Further studies are needed to explore the environmental drivers of *Blastocystis* sp. contamination. The detection of positive samples in both the dry and wet seasons suggests that *Blastocystis* can persist regardless of seasonal variations, emphasizing its resilience. Although slightly more samples were positive during the wet season than the dry season, the difference was not substantial enough to establish a significant correlation between *Blastocystis* occurrence and seasonality.

The impact could be higher than reported in this study, as cases of *Blastocystis*, a neglected parasite [59], are often underreported, highlighting the need to identify the hotspots of transmission risk. The public health implications of this study extend beyond *Blastocystis* itself, as it highlights the broader issue of waterborne diseases in resource-limited settings. The potential health impacts of *Blastocystis*, coupled with the prevalence of other pathogens in contaminated water, reinforce the urgent need for improved water quality monitoring and management. From a global health perspective, this study highlights the intersection of waterborne parasitic infections and socio-economic vulnerabilities. Improving the water quality in Guinea-Bissau will require a multifaceted approach, including the implementation of low-cost water filtration systems, education campaigns on hygiene and sanitation, and investments in sustainable infrastructure. Such interventions would not only reduce the prevalence of Blastocystis sp. but also address other waterborne pathogens of public health significance.

Despite this study’s timeframe being 2009/2010, recent research has highlighted the persistence of the key water quality indicators observed in past studies. Machado et al. [7] examined the water quality in Guinea-Bissau over a 13-year period, emphasizing the lack of intervention in WASH (water, sanitation, and hygiene) infrastructure, which has perpetuated the poor water quality and high levels of fecal contamination in drinking wells. Similarly, the study by Silveira et al. [60] showed similarities with previous studies, demonstrating that water quality issues have remained largely unchanged 14 years later. These studies reinforce the transversality of our results, suggesting that the conditions observed a decade ago may still persist today. 

Future research should address the limitations of this study. While a qPCR is highly sensitive and specific, it may fail to detect very low levels of *Blastocystis*. Metagenomic approaches could provide a more comprehensive understanding of the microbial community. Future studies should also increase dry season sampling to improve the assessments of seasonal variations, expand the sample size to enhance the generalizability of findings, and include a wider range of water sources (shallow wells vs. boreholes), as depth may influence contamination levels. Additionally, analyzing animal and clinical samples could help clarify transmission dynamics. To mitigate *Blastocystis* contamination and improve water quality in this low-income setting, sustainable interventions must consider the fragile socio-economic and political context. Longitudinal monitoring of water sources is essential for tracking seasonal trends and evaluating mitigation efforts. Long-term strategies should focus on strengthening WASH infrastructure, rehabilitating water and sanitation systems, and ensuring regular maintenance. Prioritizing the construction of deep boreholes over shallow wells, alongside routine water quality assessments and disinfection, should be key objectives [7,60]. In the short term, efforts should aim to eliminate contamination sources by relocating latrines and garbage dumps away from water sources and promoting latrine disinfection with quicklime. Additionally, transitioning from bucket-drawn wells to boreholes with pumps, installing protective barriers around water sources, and encouraging the use of narrow-mouth household water storage containers could significantly reduce contamination risks [7].

## 5. Conclusions

This study underscores the significant public health risks associated with Blastocystis sp. contamination in the rural water sources of Guinea-Bissau, where a large portion of the population relies on these supplies. The findings emphasize the urgent need for improved water management practices and increased focus on parasitic disease research in resource-limited settings. Enhancing water quality and addressing contamination sources are vital steps to protect the health and well-being of the vulnerable populations in Guinea-Bissau. In particular, targeted interventions aimed at enhancing the sanitation infrastructure, such as upgrading latrine facilities and improving waste disposal methods, are crucial. Additionally, raising community awareness about the importance of clean water and sanitation practices can empower local populations to take proactive measures. Ultimately, these actions will contribute to better health outcomes and a reduction in the burden of waterborne diseases in the region. Strengthening collaborations between local health authorities and communities will be essential to ensure sustainable improvements in water quality and public health.

## Data Availability

The original contributions presented in this study are included in the article. Further inquiries can be directed to the corresponding author.
